# HMG CoA reductase inhibitors (statins) to treat Epstein–Barr virus-driven lymphoma

**DOI:** 10.1038/sj.bjc.6602561

**Published:** 2005-04-26

**Authors:** J I Cohen

**Affiliations:** 1 Laboratory of Clinical Infectious Diseases, Medical Virology Section, National Institute of Allergy and Infectious Diseases, National Institutes of Health, Bldg. 10; Rm. 11N228, 10 Center Drive, MSC 1888, Bethesda, MD 20892, USA

**Keywords:** statin, Epstein–Barr virus, apoptosis, NF-*κ*B, lymphoma

## Abstract

While statins have been highly effective for lowering serum cholesterol and reducing the incidence of coronary events, they have multiple other effects. Certain statins block the interaction of adhesion molecules that are important for cell–cell interactions including those between EBV-transformed B cells. These same statins inhibit NF-*κ*B activation in the cells and induce apoptosis of transformed B cells. Studies in severe combined immunodeficiency mice show that simvastatin delays the development of EBV-lymphomas in these animals. These statins might be considered for the treatment of EBV-lymphomas in selected patients.

## BIOLOGY OF EPSTEIN–BARR VIRUS

Epstein–Barr virus (EBV) infects over 90% of the world's population. Most infections occur in young children and are asymptomatic or result in nonspecific symptoms. Infection of adolescents and young adults can result in infectious mononucleosis. Epstein–Barr virus is associated with a number of malignancies including Burkitt lymphoma, nasopharyngeal carcinoma, Hodgkin's disease, T-cell lymphomas, and lymphoproliferative disease in stem cell and organ transplant recipients (Cohen, 2000).

Epstein–Barr virus infects resting B lymphocytes and transforms them so that they proliferate indefinitely (Kieff, 2001). Of the nearly 100 viral proteins encoded by the genome, nine are expressed during transformation *in vitro*. These nine latency-associated proteins are the EBV nuclear antigens (EBNA-1, -2, -3A, -3B, -3C, and -LP) and the EBV latent membrane proteins (LMP-1, LMP2-A, LMP-2B). Epstein–Barr virus-transformed B cells grow in tight clumps *in vitro*.

Epstein–Barr virus gene expression differs among malignancies associated with the virus. Epstein–Barr virus-positive Burkitt lymphoma tissue shows expression of EBNA-1, but not the other latency-associated proteins (Rowe *et al*, 1987). Hodgkin's disease tissues show expression of EBNA-1, LMP-1, and LMP-2 (Deacon *et al*, 1993) while nasopharyngeal carcinoma (Fahraeus *et al*, 1988) and T-cell lymphomas (Anagnostopoulos *et al*, 1992) express EBNA-1, LMP-2 and have variable expression of LMP-1. Epstein–Barr virus lymphomas in immunocompromised persons generally show expression of each of the nine latency associated proteins. Epstein–Barr virus proteins expressed during latency may serve as targets for novel chemotherapeutic agents. While the treatment for some EBV-associated malignancies has improved in recent years, newer approaches to therapy are needed. Here, we describe the possible use of certain statins in the treatment of EBV-driven lymphomas.

## THE ROLE OF EBV LMP-1 IN ONCOGENESIS

**Figure 1:**
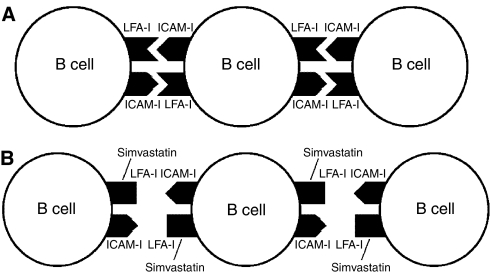
Simvastatin inhibits the interaction of LFA-1 with ICAM-1 on B cells. Epstein–Barr virus-transformed B cells express LFA-1 and ICAM-1 adhesion molecules on their surface (**A**). Simvastatin binds to the I-domain of LFA-1, inducing a conformational change that inhibits the ability of LFA-1 to bind to ICAM-1 (**B**).

LMP-1 is the latency-associated protein that has been most directly linked to oncogenesis by EBV. Expression of LMP-1 in B cells of transgenic animals results in B-cell lymphomas (Kulwichit *et al*, 1998). LMP-1 upregulates the expression of a large number of proteins on the surface of virus-infected B cells, including intercellular adhesion molecules (ICAM)-1, leucocyte function antigen 1 (LFA-1), and LFA-3 (Figure 1A) (Peng and Lundgren, 1992). Expression of LMP-1 in lymphoma cells induces clumping of the cells (Wang *et al*, 1990).

**Figure 2:**
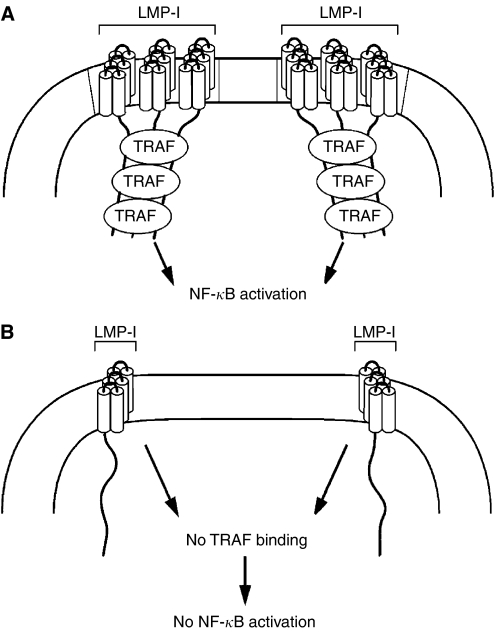
Simvastatin inhibits NF-*κ*B activation by LMP-1. Oligomerisation of LMP-1 in lipid rafts on the surface of EBV-transformed B cells recruits TRAFs to the cytoplasmic tail of LMP-1, which results in NF-*κ*B activation and inhibition of apoptosis (**A**). Simvastatin displaces LMP-1 from lipid rafts resulting in absence of NF-*κ*B activation and apoptosis of the cells (**B**).

LMP-1 acts as a functional homologue of a constitutively active form of CD40. LMP-1 oligomerises on the surface of virus-infected cells and binds to the tumour necrosis factor receptor-associated factors (TRAFs) 1,2,3 and 5, TRADD, RIP, and Janus-activated kinase (JAK) 3 (Devergne *et al*, 1996) (Figure 2A). The interaction of LMP-1 with these proteins results in activation of NF-*κ*B, c-jun N-terminal kinase, signal transducers and activators of transcription (STATs), the p38 MAP kinase pathway, and stress-activated kinases. This results in constitutive B-cell proliferation and inhibition of apoptosis. LMP-1 also interacts with p85 to activate the phosphatidylinositol 3-kinase/Akt pathway and increase cell survival (Dawson *et al*, 2003). LMP-1 upregulates several other antiapoptotic proteins including A20, Mcl-1, bcl-2, and bfl-1.

Epstein–Barr virus-associated B-cell lymphomas in humans show activation of NF-*κ*B, and LMP-1 colocalises with TRAF-1 and TRAF-3 (Liebowitz, 1998). In addition, TRAFs 1, 2, and 3 and NF-*κ*B are expressed in post-transplantation lymphoproliferative disorders (Ramalingam *et al*, 2003). Furthermore, these lesions show expression of adhesion molecules upregulated by LMP-1 including LFA-1 (Hamilton-Dutoit *et al*, 1993). Thus, LMP-1 is critical for B-cell proliferation and development of lymphomas *in vivo*.

Treatment of EBV-transformed B cells with NF-*κ*B inhibitors (e.g. I*κ*B*α* mutant, Bay11-7082) has been shown to induce apoptosis of the cells (Cahir-McFarland *et al*, 2000, 2004). We have found that treatment of EBV-transformed cells with simvastatin also inhibits NF-*κ*B and induces apoptosis of EBV-transformed B cells (Katano *et al*, 2004).

## STATINS: HMG COA REDUCTASE INHIBITORS

Statins inhibit 3-hydroxy-3-methylglutaryl coenzyme A (HMG-CoA) reductase. Six of these compounds, atorvastatin, fluvastatin, lovastatin, pravastatin, rosuvastatin, and simvastatin, are approved by the FDA for use in humans. Each of these compounds is used to treat elevated serum cholesterol. HMG-CoA reductase catalyses the conversion of HMG-CoA to mevalonate, which ultimately leads to synthesis of cholesterol. Therefore, statins reduce the level of mevalonate with a subsequent reduction in cholesterol.

Statins have a number of other activities related to their inhibition of HMG-CoA reductase (reviewed in Raggatt and Partridge, 2002). Mevalonate is a precursor for isoprenoids, including geranyl pyrophosphate and farnesyl pyrophosphate. Statins reduce the levels of these compounds. Post-translational modification of proteins by farnesylation or geranylgeranylation results in their association with cell membranes and activation (reviewed in Bellosta *et al*, 2000). These modified proteins include members of the nuclear laminin family, ras, inositol triphosphate 5-phosphatase, which are farnesylated, and Rho, Rac, cdc42, Rab, Rap, and G-proteins, which are geranylgeranylated. These changes have pleotrophic effects including inhibition of smooth muscle proliferation (Corsini *et al*, 1993), inhibition of MHC class II complexes on antigen-presenting cells (Kwak *et al*, 2000), increase in bone morphogenetic protein (Mundy *et al*, 1999), suppression of T- and B-cell responses (Kurakata *et al*, 1996), reduced NK cell activity (Hillyard *et al*, 2004), reduced synthesis of chemokines (Waehre *et al*, 2003), and growth arrest of certain transformed cells (Graaf *et al*, 2004). These effects can be reversed with the addition of mevalonate.

Certain statins have activities that are unrelated to their inhibition of HMG-CoA reductase. Kallen *et al* (1999) showed that lovastatin binds to the I-domain of LFA-1 and blocks its interaction with ICAM-1. LFA-1 (*α*_L_*β*_2_ integrin) is an adhesion molecule that promotes diapedesis of leucocytes across the endothelium and is a costimulatory molecule on activated T cells. The I-domain of LFA-1 is separate from the site of its binding to its ligand ICAM-1. Weitz-Schmidt *et al* (2001) subsequently showed that simvastatin, mevastatin as well as lovastatin (but not pravastatin) can bind to LFA-1 and block its binding to ICAM-1. This interaction is independent of HMG-CoA and is not reversed by mevalonate. Simvastatin and lovastatin concentrations of ∼10 *μ*M are required to block these interactions; this is in contrast to the nanomolar concentrations required to inhibit HMG-CoA. A synthetic statin (LFA703) which lacks HMGCoA reductase inhibitory activity, but which has increased LFA-1 binding activity, blocks LFA-1-induced costimulation of T cells and suppresses the inflammatory response to thioglycollate in a mouse model of peritonitis.

Statins have been shown to affect replication of HIV. HIV virions have ICAM-1 on their surface, which can bind to LFA-1 on target cells and enhance virus attachment (Fortin *et al*, 1997). Lovastatin inhibits replication of HIV by inhibiting the interaction of ICAM-1 on virions with LFA-1 on the surface of target cells (Giguere and Tremblay, 2004). Lovastatin inhibits HIV-induced Rho GTPase activation, which is important for HIV infection of cells (del Real *et al*, 2004). Entry into and exit from HIV-infected cells is blocked by lovastatin and this effect can be reversed by treatment with mevalonate. Treatment of HIV-infected severe combined immunodeficiency (SCID)-hu mice or humans with lovastatin results in a reduction in HIV RNA loads and increased CD4+ T-cell counts.

## SIMVASTATIN INDUCES APOPTOSIS OF EBV- TRANSFORMED B CELLS

Treatment of EBV-transformed B cells with ⩾2 *μ*M of simvastatin, atorvastatin, or lovastatin resulted in dissociation of cell clumps and death beginning 5 days after treatment with the drug (Katano *et al*, 2004). Cell death induced by simvastatin was due to apoptosis as demonstrated by detection of fragmented DNA.

While simvastatin induced cell death in EBV-positive Burkitt lymphoma cells (such as Akata, Mutu-1, Mutu-3, and P3HR-1 cells), EBV-negative B cells, and EBV-negative T cells, the concentration of simvastatin required to kill these cells (⩾4 *μ*M) was higher than for cells transformed with EBV *in vitro*.

Simvastatin and lovastatin block the interaction of LFA-1 with ICAM-1, while pravastatin does not. In contrast to apoptosis of EBV-transformed B cells induced by simvastatin, treatment of these cells with up to 16 *μ*M of pravastatin did not dissociate cell clumps or induce cell death (Katano *et al*, 2004). In addition, antibody to LFA-1, which can activate lymphocytes (Perez *et al*, 2003), prevented cell death induced by simvastatin.

### Simvastatin displaces LMP-1 from lipid rafts and inhibits NF-*κ*B

LMP-1 is present in lipid rafts on the cell membrane (Higuchi *et al*, 2001). Lipid rafts are microdomains in the membrane that are rich in cholesterol and sphingolipids and are resistant to detergent extraction. They are important for signal transduction in B cells by CD40 or by immunoglobulin on the surface of the cells. The carboxy terminal domain of LMP-1 is activated when targeted to lipid rafts where it induces signalling and activation of NF-*κ*B-mediated transcription (Kaykas *et al*, 2001). A mutation in one of the transmembrane domains of EBV LMP-1 results in lack of LMP-1 localisation to rafts, failure to bind TRAF 3, and loss of activation of NF-*κ*B (Yasui *et al*, 2004). Treatment of EBV-transformed B cells with 2 *μ*M simvastatin for 3 days reduced the amount of LMP-1 present in lipid rafts by 85% (Katano *et al*, 2004). While this effect might have been attributable to the depletion of cholesterol by the statin, it occurred at a relatively low level of simvastatin and treatment of cells with 8 *μ*M pravastatin did not result in a reduced amount of LMP-1 in rafts. Treatment of EBV-transformed B cells with 2 *μ*M simvastatin for 3 days inhibited NF-*κ*B activation. Cells treated with 8 *μ*M pravastatin did show reduced activation of NF-*κ*B.

Since LMP-1 also activates the phosphatidylinositol 3-kinase/Akt pathway (Dawson *et al*, 2003), displacement of LMP-1 from lipid rafts may reduce activation of this pathway and inhibit survival of EBV-transformed B cells.

While LMP-2 has also been shown to be present in lipid rafts (Dykstra *et al*, 2001) and simvastatin might have an effect on LMP-2, the observation that LMP-2 is dispensable for B-cell transformation by EBV (Speck *et al*, 1999), suggests that the effect of simvastatin in killing transformed B cells is unlikely to be mediated through LMP2.

### Simvastatin-induced apoptosis: inhibition of NF-*κ*B *vs* inhibition of adhesion molecule interactions

The effects of simvastatin on EBV-transformed B cells cells could be due the ability of the drug to block adhesion molecule interactions on the surface of B cells, or to displace LMP-1 from rafts and inhibit NF-*κ*B (Figures 1B and 2B). Examination of multiple EBV-positive and EBV-negative cell lines showed that induction of cell death by simvastatin correlated best with expression of LMP-1 and activated NF-*κ*B in the cells prior to treatment with drug (Katano *et al*, 2004). Cells expressing the highest levels of LMP-1 and NF-*κ*B (EBV-transformed B cells) were most susceptible to simvastatin-induced cell death; one cell line (Mutu-3 Burkitt lymphoma cells) expressing lower, but detectable levels of LMP-1 and NF-*κ*B showed an intermediate sensitivity to death by simvastatin. However, other EBV-positive cells (Akata, P3HR-1, and Mutu-1 Burkitt lymphoma cells) that expressed low levels of NF-*κ*B and no detectable LMP-1 were much less sensitive to simvastatin. One of these latter cell lines (P3HR-1) expressed levels of LFA-1 and ICAM-1 that were similar to those in EBV-transformed B cells. Taken together, these finding suggest that inhibition of NF-*κ*B by simvastatin may be more important than its ability to block the interaction of LFA-1 with ICAM-1.

### Studies of simvastatin in an animal model of EBV lymphoma

Intraperitoneal injection of EBV-transformed B cells into SCID mice results in development of EBV-positive lymphomas that resemble the tumours seen in immunosuppressed persons (Rowe *et al*, 1991). These tumours show a pattern of gene expression similar to that in patients with EBV lymphoproliferative disease with EBNAs, LMP-1, and adhesion molecules detected in the tumours.

**Figure 3:**
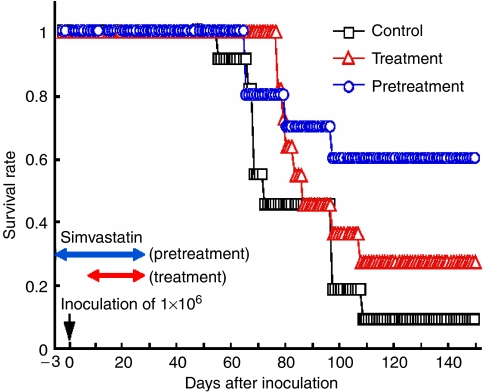
Simvastatin increases survival of SCID mice inoculated with EBV-transformed B cells. Mice treated with simvastatin before inoculation with EBV-transformed B cells have improved survival (*P*<0.04) compared with untreated mice. Mice treated with simvastatin after inoculation with virus-transformed B cells show a trend (not statistically significant) toward improved survival. Reproduced with permission from Katano *et al* (2004).

Oral treatment of SCID mice with simvastatin beginning 3 days prior to injection with EBV-transformed B cells resulted in a statistically significant improvement in survival rate compared to animals not given the drug (Figure 3) (Katano *et al*, 2004). While there was a trend for longer survival for mice treated with simvastatin beginning 7 days after injection with EBV-transformed B cells, the difference with untreated mice was not statistically significant. Some tumours from mice that were treated with simvastatin showed downregulation of LFA-1 on their surface, compared with the EBV-transformed B cells that had been used to inject the animals.

The dose of simvastatin used to treat these mice (250 mg kg^−1^ day^−1^) is estimated to result in serum levels that would be 4 to 8 times that of humans receiving the maximum dose of simvastatin (80 mg day^−1^) used to lower serum cholesterol. However, similar high serum levels have been achieved in humans treated with large doses of statins in cancer therapy trials (see below).

## STATINS FOR TREATMENT OF OTHER TUMOURS

Statins have been shown to induce apoptosis in several proliferating tumour cell lines, including certain leukaemia, lymphoma, astrocytoma, pancreatic carcinoma, and neuroblastoma cell lines (reviewed in Wong *et al*, 2002). This effect is due to inhibition of HMG-CoA reductase since it can be inhibited by mevalonate. Lovastatin reduced viability of EBV-positive or EBV-negative Burkitt lymphoma cells by >75% due to inhibition of geranylgeranylation (van de Donk *et al*, 2003). Statins have been used in animal models and have reduced the tumour burden associated with melanoma, hepatoma, neuroblastoma, pancreatic cancer, and lung cancer (reviewed in Wong *et al*, 2002). In other animal models, statins have been used as adjunctive therapy in combination with cytotoxic agents in mouse models including carmustine for melanoma and with doxorubicin for lung cancer.

### Clinical studies of statins

A number of studies have tested the role of oral statin therapy in patients with tumours (reviewed in Wong *et al*, 2002). Lovastatin given in doses ranging from 2 to 45 mg kg^−1^ day^−1^ for 1 out of every 4 weeks was used to treat a variety of tumours including astrocytoma, glioblastoma, and prostate, breast, ovarian, and lung cancer (Thibault *et al*, 1996). Doses of 25 mg kg^−1^ day^−1^ for 7 days were well tolerated. Serum levels of the drug ranged from 0.1 to 3.9 *μ*M. One patient with an astrocytoma had a minor response to therapy. Since mice metabolise statins more rapidly than humans, a dose of 25 mg kg^−1^ day^−1^ is comparable to ∼250 mg kg^−1^ day^−1^ in mice.

While large doses of statins have been used in patients with cancer, might standard doses of statins prevent development of haematologic malignancies? Large trials of simvastatin for the prevention of coronary events have evaluated the incidence of cancer in study recipients. No significant decrease in the overall incidence of cancer or of haematologic malignancies was noted in the MRC/BHF Heart Protection Study involving 20 536 patients randomised to 40 mg of simvastatin per day or placebo for a median of 5 years (Heart Protection Study Group, 2002). Similarly, there was no significant decrease in the incidence or mortality for cancer, or for lymphatic or haematopoeitic malignancies in the Scandinavian Simvastatin Survival Study in which 4444 patients were randomised to simvastatin (20–40 mg per day) *vs* placebo for a median of 5.4 years (Strandberg *et al*, 2004). Thus, simvastatin at the standard doses to prevent coronary events did not have a significant effect on the risk of haematologic cancers.

## TREATMENT OF EBV-DRIVEN LYMPHOMA

EBV lymphomas occur in immunocompromised patients such as organ or stem cell transplant recipients, patients with HIV, or patients with congenital immunodeficiencies. These tumours generally express each of the EBV latency proteins and the tumours are driven by these viral proteins. Central nervous system lymphomas account for 20% of lymphomas in AIDS patients and express EBV LMP-1 and other latency proteins (MacMahon *et al*, 1991). Immunoblastic lymphomas account for about 60% of cases of lymphoma in patients with AIDS and usually express EBV LMP-1 and other latency proteins (Hamilton-Dutoit *et al*, 1993). These tumours generally occur in AIDS patients late in the course of disease when CD4+ T cell numbers are low.

Tumours in immunocompromised patients can occur in lymph nodes, but frequently present at extranodal sites such as the gastrointestinal tract, central nervous system, liver, lung, bone marrow, or transplanted organ (Cohen, 2000). The tumours can be polyclonal or monoclonal and usually lack chromosomal translocations. Patients who lack immunity to EBV at the time of transplant and develop primary EBV infection after transplant are more likely to develop EBV-driven lymphomas. Transplant recipients who receive HLA-mismatched or T-cell-depleted bone marrow or infusions of antilymphocyte antibodies for rejection are also at increased risk for development of lymphomas. Epstein–Barr virus viral DNA is often elevated in peripheral blood mononuclear cells of these patients prior to, and at the onset of lymphoma, indicative of the EBV-driven B-cell proliferation. Patients with these tumours have impaired T-cell immunity to EBV resulting in failure to regulate EBV-driven B-cell proliferation.

Treatment for these lymphomas includes reduction in immunosuppression when possible. Resection of localised lesions, especially in the gastrointestinal tract has been effective in some patients. Monoclonal anti-CD20 antibody (rituximab) results in remissions in about 50% of patients. Interferon-*α* has been effective in some patients, but may increase the risk of rejection of the transplanted organ. Lymphomas in stem cell transplant recipients are usually of donor origin; infusions of donor T cells (which are HLA-matched) have been effective in many cases of EBV lymphoma in these patients. Lymphomas in organ transplant recipients are usually recipient in origin; infusions of autologous or HLA-matched T cells have been effective. Radiation therapy, especially for central nervous system lesions, and cytotoxic chemotherapy are used for refractory cases. The latter two therapies are frequently used for lymphomas in AIDS patients whose immune systems are less responsive to immunologic-based therapies.

Statins may have a role as adjunctive therapy in some patients with EBV-driven lymphomas. These might include stem cell transplant recipients in whom donor T cells are not available, organ transplant recipients whose lymphomas are not responsive to reduction of immunosuppressive therapy and in whom HLA-matched T cells are not available. In these settings, statins might be used in combination with other therapies (e.g. rituximab or interferon-*α*) which by themselves result in remissions in about 50% of patients. Statins might be tried in AIDS patients with EBV-driven lymphomas, especially those with very low CD4T cell counts who tend to respond less well to chemotherapy. Statins might also be considered for patients with EBV-positive Hodgkin's disease who are refractory to chemotherapy and radiation therapy. Tumours from these patients usually express LMP-1 as well as adhesion molecules (Sandvej *et al*, 1993), and statins might reduce the viability of the tumour cells. While nasopharyngeal carcinoma and T-cell lymphomas frequently express LMP-1, the tumours show more variable expression of the protein and therefore might be less susceptible to statins. Finally, Burkitt lymphomas do not express LMP-1 and therefore would be unlikely to respond to statins at doses that are effective for cells transformed with EBV *in vitro*.

## CONCLUSIONS

Identification of signalling pathways for EBV-mediated transformation has helped to identify new targets and potential treatments for these tumours. Certain statins have been shown to inhibit the interactions of adhesion molecules and block NF-*κ*B activation in EBV-transformed cells, resulting in apoptosis. Simvastatin delays the development of EBV-lymphomas in SCID mice inoculated with EBV-transformed B cells. The dose of statin needed to induce apoptosis is much higher than that required for lowering serum cholesterol, but such doses have been tolerated by patients in clinical trials. Statins may have a role in the treatment of EBV-driven lymphomas, most likely as part of combination therapy for these lymphomas.
